# Co-occurrence of TCF3-PBX1 gene fusion, and chromosomal aberration in a pediatric pre-B cell acute lymphoblastic leukemia with clitoris swelling: A case report and literature review

**DOI:** 10.1097/MD.0000000000024802

**Published:** 2021-02-26

**Authors:** Guo-qian He, Xia Guo, Ming-yan Jiang, Rong-rong Xu, Yi-ling Dai, Lili Luo, Ju Gao

**Affiliations:** a Key Laboratory of Birth Defects and Related Diseases of Women and Children, Ministry of Education; b Department of Pediatrics; c Department of Radiology, West China Second University Hospital, Sichuan University, Chengdu, Sichuan, P.R. China.

**Keywords:** acute lymphoblastic leukemia, case report, chromosome, clitoris, swelling, TCF3-PBX1 fusion

## Abstract

**Rationale:**

Clitoris swelling as the initial clinical presentation of acute lymphoblastic leukemia (ALL) is extremely rare. These patients may be misdiagnosed with acute myeloid leukemia or solid tumor, and the main treatment can also be delayed.

**Patient concerns:**

A 2.10-year-old girl was referred to the pediatric surgery clinic with a worsening onset of clitoris swellings. The patient was afebrile and well appearing. Multiple retroperitoneal mass were confirmed by computed tomography (CT) and high serum neuron-specific enolase level was high. She was scheduled for an abdominal biopsy from the retroperitoneal mass suspicious of neuroblastoma.

**Diagnoses:**

The child was eventually diagnosed as having precursor B cell ALL with central nervous system involved, with TCF3-PBX1 fusion gene and additional chromosomal aberrations, based on examinations of the bone marrow and brain magnetic resonance imaging.

**Interventions:**

Before the diagnosis of leukemia, the patient was given symptomatic treatment for 1 week. She was treated with chemotherapy in accordance with the Chinese Children's Cancer Group protocol 2015 after confirmed diagnosis.

**Outcomes:**

After induction chemotherapy for ALL, although the girl had transiently clinical remission, the bone marrow aspirate indicated a poor outcome. Our patient discontinued treatment and discharged. From literature review, there was only 1 case of in acute myeloid leukemia with clitoris swelling as the initial symptom.

**Lessons:**

The clinical symptoms of ALL with clitoris swelling are not typical, with a high rate of misdiagnosis. When the cause of clitoris swelling is unknown, ALL should be considered. Bone marrow aspiration must be done before doing a more invasive investigation like biopsy.

## 1 Introduction

Pediatric B-cell acute lymphoblastic leukemia (ALL) is the most common malignant disease in children.^[1]^ It involves malignant transformation and proliferation of immature lymphoid cells in the bone marrow, blood, or extramedullary sites.^[2,3]^ ALL is clinically characterized by high invasiveness like fever, bleeding, bone pains, lymphadenopathy or hepatosplenomegaly, and extramedullary invasion.^[4,5]^ However, clinical palpable soft tissue swelling is rarely seen in ALL at presentation, which is more likely present in acute myeloid leukemia (AML) or solid tumors. Moreover, ALL with clitoris swelling as the initial symptom is very rare. The association of clitoris swelling has been reported in only one case of AML.^[6]^ However, it had not been reported in ALL. It may be prone to misdiagnosed and miss prompt treatment. Here, we report about a child with an abrupt onset of clitoris swelling as the initial symptom. This is an unusual case with high neuron-specific enolase (NSE). The patient was finally diagnosed as having B-cell precursor ALL (BCP-ALL) harboring TCF3-PBX1 and additional chromosomal aberrations after the misdiagnosis of hermaphroditism or neuroblastoma for 2 months at a local. In addition, clinical data were retrospectively analyzed, and the literature was reviewed. Thus, to highlight leukemia should be considered in a child presenting with clitoris swelling.

## 2 Case description

This study was approved by the Ethics Committee of the West China Second University Hospital and written informed consent was obtained from parents of the child.

A 2.10-year-old girl was admitted to our hospital with presentation of an onset of clitoris swelling for 2 months, pallor for 3 days, and fever for 1 day. Two months before admission, the child had no obvious reason for clitoris swelling and did not have fever. She had no complaints of local trauma, fever, pain, abdominal distention, lymphadenopathy, weight loss, or dysuria. The local hospital diagnosed the condition as hermaphroditism. However, the cortisol level and sex hormone level were normal at that time point. The karyotype was normal (46, XX). Clitoris ultrasonography showed a soft 2.6 × 1.3 cm swelling structure like cavernous body. The child had received dietary regulation for stopping taking 2 eggs for 1 week, after which the clitoris swelling had transiently diminished in size. One month before admission, the child had inappetence and vomiting for 1 day, presented to the pediatric surgery clinic (the West China University Hospital of Sichuan University), and routine blood test results revealed elevated white blood cell (WBC) count of 16.68 × 10^9^/L (3.5–9.5 × 10^9^/L), with slightly decreased hemoglobin levels of 93 g/L (115–150 g/L), and platelet (PLT) count of 35 × 10^9^/L (100–300 × 10^9^/L), without unidentified cells. C-reactive protein (CRP) levels increased to 5.87 mg/L (<5 mg/L). In addition, the serum NSE level was 130.5 ng/mL (<15 ng/mL) and alpha-fetoprotein (AFP) was normal. Computed tomography (CT) showed clitorism, multiple mass on the pancreas with the largest lesion at the head, on the right adrenal gland, and on the left kidney, and probable metastasis in inguinal lymph nodes. She was scheduled for an abdominal biopsy from the retroperitoneal mass suspicious of neuroblastoma. Frozen section revealed a small round blue cell tumor. The symptomatic treatment was started with anti-infection for 1 week, with no additional chemotherapy. The swelling on clitoris had transiently diminished in size.

**Figure 1:**
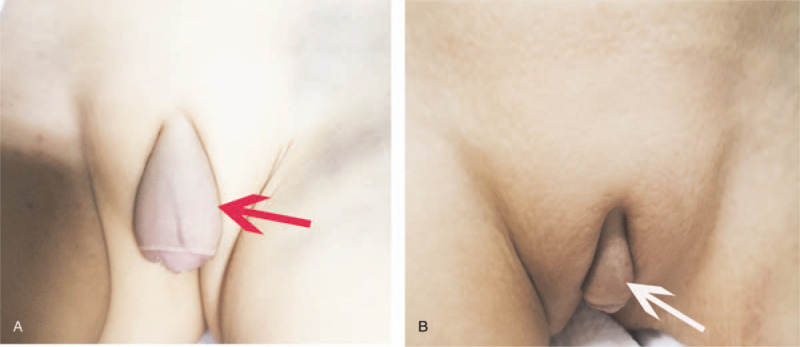
A. The thickened and hyperemic skin of the clitoris extended to the groin region (red arrow). B. The clinical of the clitoris was improved after treatment (white arrow).

The patient was referred to our hospital for further treatment of retroperitoneal mass suspicious of neuroblastoma. Results of the physical examination of admission were as follows: temperature, 36.6 °C; respiration rate, 48 beats/min; heart rate, 131 beats/min; blood pressure, 83/43 mm Hg; and weight, 13 kg. She had clitoris swelling (Fig. 1A), lower gums swelling and invagination, a 3 × 4 cm mass on the right forehead, and hepatosplenomegaly on exam (live was palpable 12 cm and spleen were palpable 9 cm below the costal margin). The patient's spirit was not as good, and her complexion was not as ruddy, slight enlargement of the inguinal lymph nodes, and petechiae in the skin. Auxiliary examination results were as follows: routine blood examinations: WBC 6.7 × 10^9^/L (3.6–13 × 10^9^/L), N, 11.0%; L, 39.0%; MN 2.0%; hemoglobin (HGB), 82 g/L; PLT, 29 × 10^9^/L; and CRP 12.0 mg/L, and abnormal cells were detected (46%). She had hyperuricemia 787 μmol/L (184–464 μmol/L), high serum lactate dehydrogenase level 7793 U/L (120–246 U/L), and high ferritin level 778.2 ng/mL (10–291 ng/mL). Serum NSE level was >370 ng/mL (<15 ng/mL). The results of blood EB virus, TORCH, and blood transfusion immunoassay were normal. Endocrine studies suggest that the girl had hyperprolastinemia >200 ng/mL (2.8–29.2 ng/mL), but normal levels of morning ACTH, morning cortisol, thyroid function, serum adrenocorticotropic hormone, sex hormone, human chorionic gonadotropin, cortisol, and anti-mullerian hormone.

**Figure 2:**
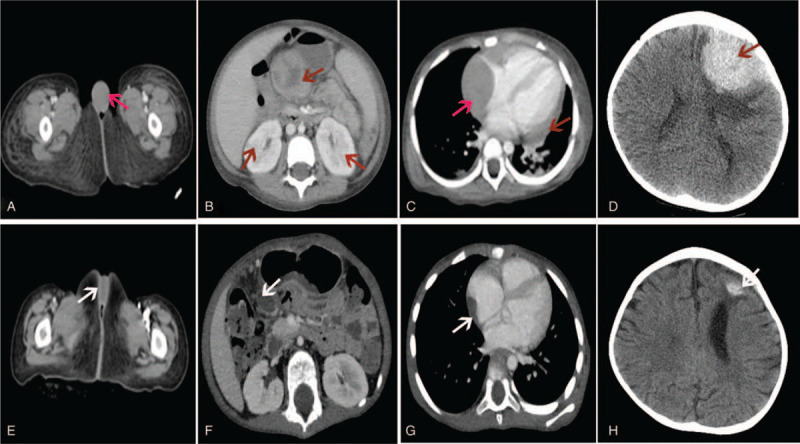
The findings in the clitoris, chest, abdomen, and brain before and after chemotherapy treatment. The location of the lesion was shown by the arrow. A. CT showed the mass in the clitoris. B. Abdomen CT showed an enlarged liver and spleen, multiple mass in the both kidneys. The enhancement CT scan of abdominal indicated a cystic mass on the neck of the pancreas which protruded outside the outline of the pancreas (red arrows). C. A chest CT showed a round soft tissue shadow (3.8 × 3.3 cm, red arrows) around the right atrium, with no obvious enhancement. The lesion compression was near the right atrium, and there is with few of pericardial effusion. D. The head CT showed a circular high-density shadow on the left forehead, with a size of about 5.3 × 4.2 cm and a circular low-density shadow around it. The lesion is showing mild enhancement. The lesions located in the clitoris (E), abdomen (F), chest (G), and head (H) were significantly reduced (white arrow). CT = computed tomography.

**Figure 3:**
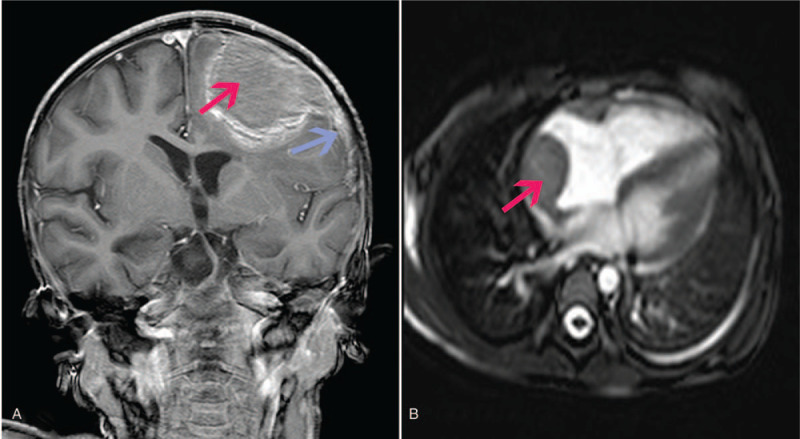
A. Brain MRI enhancement imaging showed mild enhancement with a wide base connected to the meninges (blue arrow). The MRI showed a round-like mixed signal shadow on the left frontal and parietal part on the sagittal position, mainly presented with T2 equal signal, and the surrounding parenchyma was slightly compressed (red arrow). B. MRI of heart also showed an isodense on T1WI or T2WI on the top of the right atrium. MRI = magnetic resonance imaging.

A repeated CT scan of chest and abdomen showed an enlarged liver and spleen, multiple mass on the clitoris (Fig. 2A), the neck of the pancreas (Fig. 2B), the right adrenal gland, both kidneys (Fig. 2B), the right atrium (Fig. 2C), and left ovarium. Heart Doppler ultrasound showed that right atrial enlargement (RA = 39 mm), a well defined periphery with a slightly hypoechoic (4.0 × 2.9 × 3.5 cm) in the right atrium. MRI of heart (Fig. 3B) also confirmed the lesions. A cerebral spinal fluid analysis was sent for and it showed no malignant cells. However, CT scan images and magnetic resonance imaging (MRI) of the brain revealed a well-defined enhanced and largest lesion at the left forehead (Figs. 2D and 3A) and mild enhancement with a wide base connected to the meninges (Fig. 3A), indicated the central nervous system involvement.

**Figure 4:**
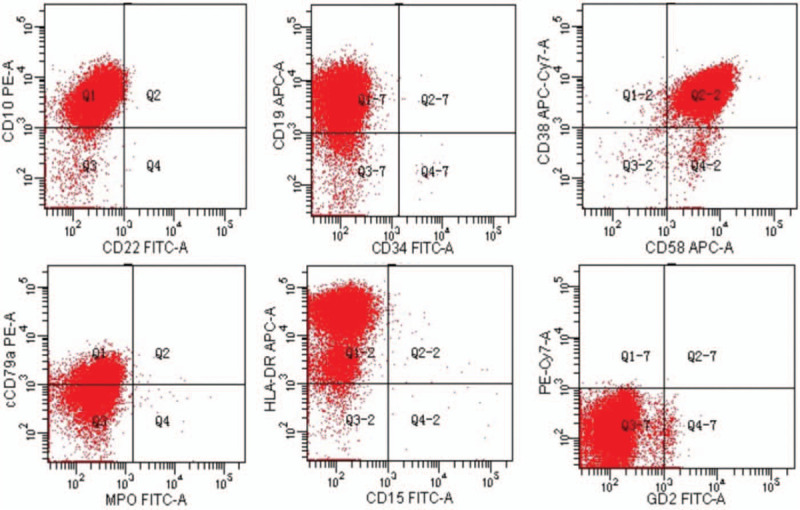
The aberrant B-lymphoblast population in bone marrow aspirates on flow cytometry. Flow plots on the top panel prior to chemotherapy (left to right) show the expression pattern of CD10, CD19, CD38/58, Ccd79, and HLA-DR on the aberrant B-lymphoblast population.

**Figure 5:**
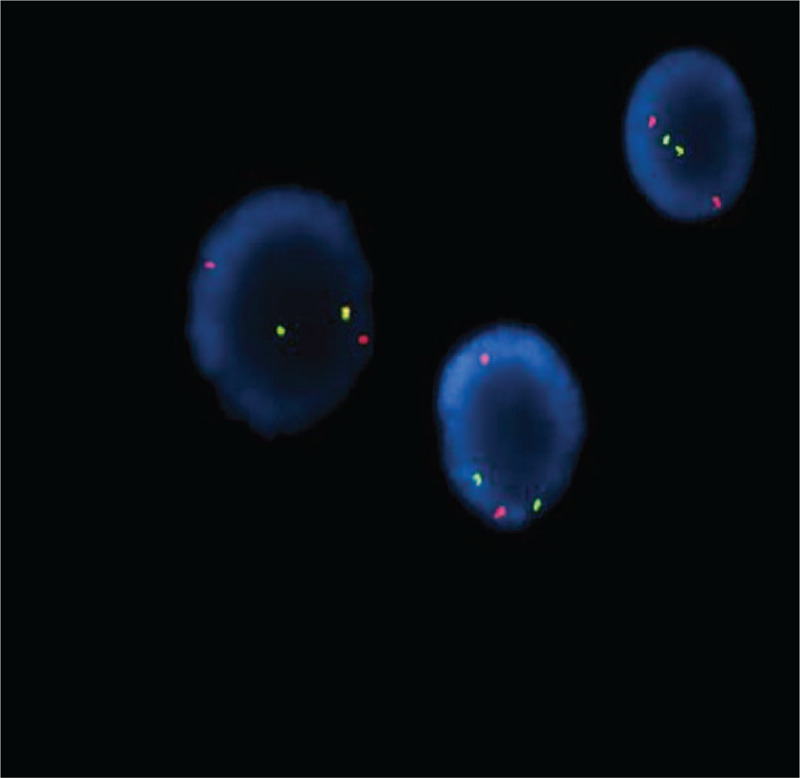
The flurorescence in situ hybridization (FISH) analysis used a dual-color break apart probe, showed the presence of the TCF3/PBX1 (E2A-PBX1) fusion gene. A positive TCF3 rearrangement showed one split signal (1 Red, 1 Green).

**Figure 6:**
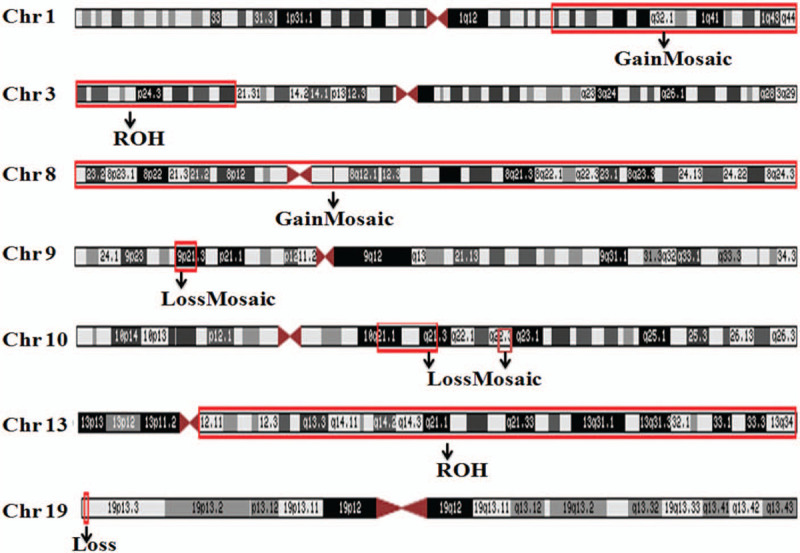
A chromosome analysis on peripheral-blood using chromosome microarray analysis (CMA) showed 8 chromosomal abnormal (LossMosaic: copy number loss, GainMosaic: copy number gain, ROH: copy number normal).

On admission, the diagnosis was neuroblastoma or lekemia. Because of neuroblastoma with bone marrow invasion could not be excluded. A comprehensive immunohistochemistry panel was performed in the retroperitoneal mass, as well as bone marrow (BM) aspirate smears were done for further investigation. Pathology showed precursor lymphoid tissue tumors: a B-cell lymphoblastic lymphoma or leukemia. The tumor was positive for CD20, CD79a, CD99 (individual), CD10, PAX-5, TdT (partly), P53 (<5%), bcl-2 (>90%), and C-myc (5–10%). CD3, CD5, CD117, CD30, MPO, CD21, CD30, and EBER1/2 were all negative. Further molecular testing by PCR and Genescan demonstrated IgH and IgK cloning peak amplification. BM aspirate showed 94% lymphoblasts. The immunophenptype analysis by flow cytometry analysis showed CD10 positive cells with expression of cCD79a, CD19, CD38, CD58, HLA-DR (Fig. 4). Precursor B-cell ALL was diagnosed. Additionally, flurorescence in situ hybridization (FISH) detected TCF3-PBX1 fusion gene in 24% of interphase cells on bone marrow aspirate (Fig. 5). The minimal residual disease (MRD) analysis by Real-time qPCR showed that sLambda marker. The bone marrow cytogenetic study showed 46, XX [15] chromosomes. However, a chromosome genome-wide chip analysis (CMA) detected the 8 different chromosomal abnormalities in of chr 1, 3, 8, 9, 10, 13, and 19 (Fig. 6). Copy GainMosaic were detected at 1q23.3 and 8p23.3q24.3, and a runs of homozygosit (ROH) pattern at 3pter21.33 and 13q11qter, which were compatible with the diagnosis of ALL. Copy LossMosaic were detected at 9p22.1p21.3 and 10q21.1q21.3, which were compatible with the diagnosis of ALL. Notably, copy LossMosaic also detected at 10q22.1, which was possible compatible with the malignant blood diseases, and 19p13.3, which was an unknown clinical significance. The study Targeted sequencing of all coding exons for blood tumor-related genegs, revealed that a mutation of Coiled-Coil Domain Containing 88A (CCDC88A) on chromosome 2 (NM_018084:EXON30:c.5225C>T:p.Ala1742Val) with a frequency of 39.2%, the significance of which remains unknown in this tumor. Based on these finding, the patient was diagnosed as having precursor B-cell ALL with central infiltration and cerebral hemorrhage, positive for TCF3-PBX1 fusion gene, and additional chromosomal aberrations.

## 3 Treatment

The patient received antiinfective therapy because of pulmonary infection, symptomatic treatment because of intracranial hypertension and non-invasive ventilator therapy because of respiratory failure. According to the Chinese Children's Cancer Group protocol (CCCG-ALL-2015) based on St. Jude Total XV protocol,^[7]^ intermediate-risk ALL chemotherapy protocol was given. Chemotherapy with dexamethasone (6 mg/m^2^ per day) for 4 days was administrated simultaneously, with prednisone (45 mg/m^2^ per day, from day 5–28), daunorubicin (25 mg/m^2^ at day 5, 12), vincristine (1.5 mg/m^2^ at day 5, 12, and 19), and PEG-asparginase (2000 U/m^2^ at day 6). Due to the risk of brain herniation, the triple intrathecal therapy was initially deferred and treatments for a total of 5 doses. Treatment response was evaluated at day 19 and day 46 by morphologic criteria and flow cytometric minimal residual disease (MRD) measurements.

## 4 Outcome and follow-up

**Figure 7:**
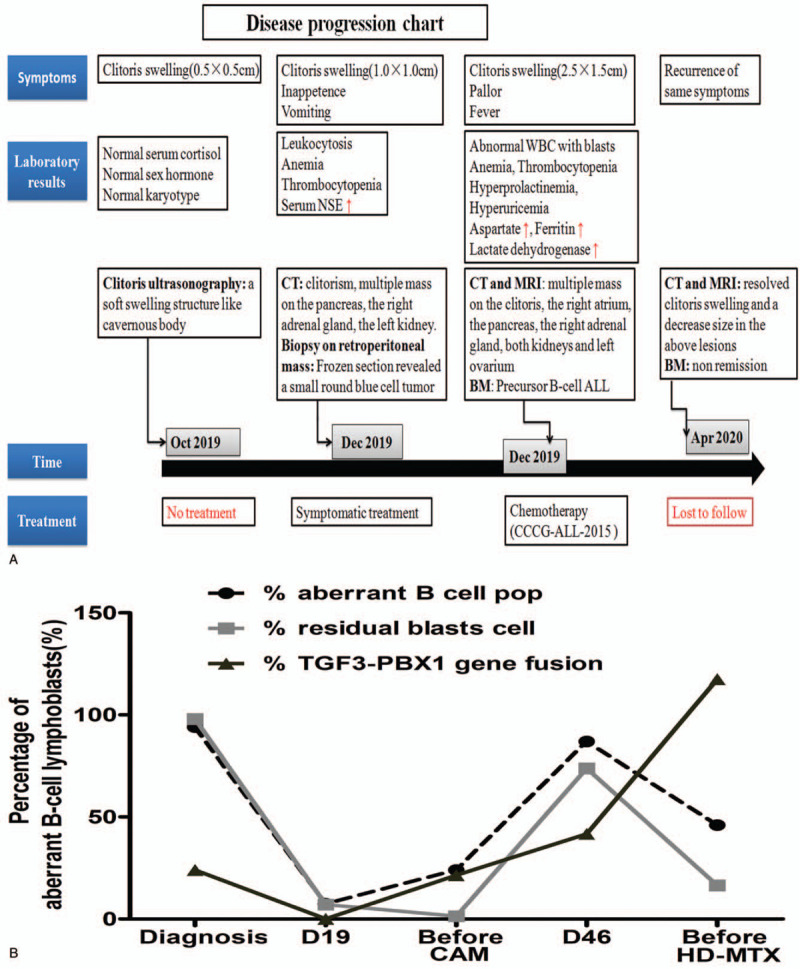
A. The timeline of clinical events and treatment of the case discussed in this case report. The unexplained clitoris swelling began in Oct 2019. Laboratory work-up revealed elevated NSE along with leukocytosis, anemia, and thrombocytopenia. An abdominal biopsy from the retroperitoneal mass was done. Frozen section revealed a small round blue cell tumor. A bone marrow evaluation showed acute lymphoid leukemia. A diagnosis of was B-cell precursor-ALL made and non-complete remission response to chemotherapy. B. Trend of molecular and cytogenetic abnormalities, including the percentage of aberrant B cell pop by bone marrow morphologic, the percentage of residual blasts cell by MRD and the molecular levels percentage of TGF3-PBX1 gene fusion by FISH at key time point. The right *y*-axis represents the percentage of aberrant B-cell lymphoblasts. The *x*-axis denotes the time points during chemotherapy at which that the molecular and cytogenetic data were obtained. MRD = minimal residual disease; MRI = magnetic resonance imaging; NSE = neuron-specific enolase.

On the 17th day of the induction chemotherapy (12 days after the first dose of PEG-asparginase), she presented with acute pancreatitis (serum lipidemia and amylase elevated). CT scan showed no lesions or edema in pancreatic parenchyma. After treatment for acute pancreatitis, she got clinical remission and went on with chemotherapy, but had not received the second PEG-asparginase at day 26. After 4 weeks of induction chemotherapy, physical examination showed a decrease in size of the clitoris swelling (Fig. 1B), and the lower gums was improved. Bone marrow aspiration at Day 19 revealed bone marrow in partly remission (7.5% of lymphoblasts and 7.09% residual blasts by MRD), and negative for TCF3-PBX1 gene fusion by FISH analyses (Fig. 7B). CT scan of the brain, chest, and abdomen showed resolved clitoris swelling and a decrease size in the left forehead, the right atrium, the pancreas, and the left ovarium (Fig. 2E–H). No lesions were found in the both kidneys and right adrenal gland.

The patient was next given twice a 3-week induction chemotherapy composed of cyclophosphamide, cytarabine, and mercaptopurine. As show in Fig. 7B, unfortunately, bone marrow aspirate before this stage treatment showed 1.35% residual blasts by MRD, but positive for TCF3-PBX1 gene fusion by FISH analysis (21.44%). Bone marrow aspirate at Day 46 revealed bone marrow in non-remission (87% of lymphoblasts and 73.87% residual blasts by MRD), and positive for TCF3-PBX1 gene fusion (21.44%). However, the repeated CT scan of brain, chest, and abdomen showed the reduced size of multiple lesions. The patient stopped on the consolidation phase of chemotherapy. Because her BM aspirate before this phase treatment showed morphologic non-remission (46% blasts cell), 16.5% residual blasts by MRD, and positive for TCF3-PBX1 gene fusion by molecular analysis (117.5%). The peripheral blood showed pancytopenia with blasts and neutropenia. Because of the low success rates of current chemotherapy for refractory ALL, the parents went for alternative treatment for their daughter and were lost to follow-up (Fig. 7A).

## 5 Discussion and review of literature

ALL is a malignant disorder of lymphoid progenitor cells, commonly presents as are hepatomegaly, splenomegaly, pallor, fever, and bruising.^[8]^ The liver, spleen, or lymph nodes are the most common sites of extramedullary involvement in ALL. ALL presenting as a soft tissue mass is very rare. The patient discussed in this study presented unusual symptom rarely reported in the literature. Clitoris swelling at presentation or in the course of ALL is rare with no case reported in the literature. Soft tissue masses are more common in AML. Only one study had reported an 11-year-old white girl with acute nonlymphocytic leukemia had a history of fever, headache, and red swelling of the clitoris.^[6]^ Although as the presence of the leukemic clitoris in ALL is not common, although rare, their significance is uncertain. The pathogenesis of the involvement of clitoris was uncertain. In this case, clitoris may be as the sites of extramedullary involvement in ALL. The involvement of the clitoris in pediatric leukemia should always be remembered either as initial symptomatology.

Moreover, in the present case, the multiple retroperitoneal mass and the elevation of serum NSE level might have led to a clinical misdiagnosis. NSE, as a useful marker for the diagnosis of neuroendocrine tumors such as neuroblastoma, is usually synthesized by neurons and neuroendocrine cells. At the initial evaluation, the patient was diagnosed with suspicious of neuroblastoma and scheduled for an abdominal biopsy from the retroperitoneal mass. However, NSE is also found in lymphocytes. Some of the hematopoietic cell lines including T-cell leukemia and Epstein-Barr virus-immortalized B lymphoblastoid cell lines produce NSE. Bone marrow examination did not support this disease. So, the abdomen multiple mass and the elevation of serum NSE level should be considered in the differential diagnosis. In case the initial workup is inconclusive, a bone marrow aspiration must be done before doing abdomen biopsy which is a more invasive and risky procedure.

Extramedullary deposits in central nervous system (CNS) or testiculus are less common in ALL.^[9,10]^ Involvement of the CNS is seen in 2% to 5% of ALL patients and testicular disease in 1% of patients.^[3,11,12]^ ALL is also characterized by genetic and epigenetic aberrations including chromosomal translocations, which result in aberrant fusion genes.^[1,13]^ TCF3-PBX1 fusion gene is well-known chromosomal abnormalities in pediatric B-cell precursor (BCP)-ALL. Patients with TCF3-PBX1 fusion gene expression usually had a more aggressive disease course, were found to have higher white blood cell counts at diagnosis or increased CNS relapse.^[14]^ Chromosomal abnormalities and expression of defective gene clones at diagnosis are used as the main prognostic factors for leukemia.^[15,16]^ The patient was diagnosed with pre-B-cell acute lymphoblastic leukemia with cytogenetics showing chromosomal translocation of t(1;19)(q23;p13.3), which results in the TCF3-PBX1 fusion gene. The latter is one of the most frequent rearrangements observed in 3% to 5% B cell ALL and reported that specific for pre-B ALL.^[14,17–19]^ The TCF3-PBX1 gene is one of the HOX DNA-binding cofactors.^[20,21]^ HOX genes were reported that can result in altered self-renewal and differentiation of hematopoietic stem cells. Most cases have a typical immunophenotype with homogeneous expression of CD19, CD10, CD9, and complete absence of CD34.^[18,22,23]^ Moreover, the TCF3-PBX1 gene with is associated with known clinical high risk factors, such as elevated white blood cell count, high serum lactate dehydrogenase levels, and central nervous system involvement.^[15,18,24,25]^ However, in our study, this patient did not present with hyperleukocytosis or central nervous system symptoms at onset. In ALL, children with TCF3-PBX1 fusion usually had a more aggressive disease course and a poor long-term outcome under standard treatment.^[23,26]^ In one study, allogeneic hematopoietic stem cell transplantation (HSCT) was able to overcome the poor outcomes of these patients with TCF3-PBX1.^[27]^ However, it must be offered to patients during the first morphological complete remission (CR). In our study, this patient did not have morphological complete remission throughout the treatment procedure. A study showed that anti-CD19 CAR-T cells therapy with a remarkable MRD eradicating ability might be an effective option for patients with relapsed and refractory TCF3-PBX1 positive B-ALL.^[28,29]^ In our study, the low success rates of the standard chemotherapy based on CCCG-ALL-2015 was observed. The anti-CD19 CAR-T cells therapy may be an optional choice for this girl.

In this case, the tumor whole exome sequencing showed that a mutation of CCDC88A on chromosome 2 was observed, the significance of which remains unknown in leukemia. CCDC88A encodes Girdin, a member of the family of coiled-coil domain containing proteins. Physiologically, Girdin is mainly expressed in the nervous system, tendons, heart valves, and skeletal muscle,^[30]^ and highly expressed in many types of cancer,^[31]^ such as edema, hypsarrhythmia and optic atrophy (PEHO) syndrome,^[32]^ Glioma,^[33]^ and pancreatic cancer.^[30,34]^ There were no relevant literatures on leukemia reported. In our study, the child had pancreatic involvement. However, the child did not have common pathologic changes, such as pancreatic cancer or PEHO. Girdin was an actin-binding protein identified as a novel substrate of AKT, and enhances AKT signaling by mediating phosphatidylinositol 3-kinase (PI3-K)/AKT signaling pathway.^[34,35]^ The PI3K/AKT signaling pathways are activated in acute myeloid leukemia.^[36]^ In addition, Girdin also affects the proliferation and metastasis of tumor cells, malignant angiogenesis, and is negatively correlated with the prognosis of cancer.^[37]^ Although the mechanism responsible for CCDC88A involvement in leukemia response is unclear, given the role of AKT in the development of leukemia, it was not surprising that this gene might play a role in variable response to treatment of ALL. Further mechanistic studies of these genes as well as additional association studies in patients with leukemia are needed.

In conclusion, when reviewing the whole process of diagnosis and treatment, we believe that bone marrow examination should be done early for child with rapid onset of soft tissue swelling, to avoid clinical misdiagnosis.

## Acknowledgments

The authors thank the patient's parents for providing permission to use the information of their children.

## Author contributions

**Data curation:** Guo-qian He, Ming-yan Jiang, Yi-ling Dai, Li-li Luo.

**Formal analysis:** Yi-ling Dai.

**Funding acquisition:** Li-li Luo, Ju Gao, Xia Guo.

**Resources:** Guo-qian He, Rong-rong Xu.

**Writing – original draft:** Guo-qian He.

**Writing – review & editing:** Ju Gao, Xia Guo.
